# A Year of Medicine in Buffalo

**Published:** 1898-01

**Authors:** 


A Monthly Review of Medicine and Surgery.

*EDITORS:*

THOMAS LOTHROP, M. D. - - WM. WARREN POTTER, M. D.

All communications, whether of a literary or business nature, should be addressed to the managing editor: 284 Franklin Street, Buffalo, N. Y.

Vol. XXXVII. JANUARY, 1898. No. 6.

### A YEAR OF MEDICINE IN BUFFALO.

THE year 1897 having just terminated, a résumé of medical affairs would seem appropriate. Such reviews have historic interest, serving to group within a single article such data of interest as may come within the purview of the historian of the future.

With the advent of the year came the new appointments in the health department. Mayor Jewett reappointed Dr. Ernest Wende as health commissioner for a term of five years, to succeed himself. Dr. Wende appointed as deputy commissioner Dr. Walter D. Greene, a prominent physician, also for the term of five years. Dr. John A. Pettit retired as deputy health commissioner after five years of honored service, on account of the pressing demands of his practice. Dr. Pettit has been a prominent practitioner in Buffalo for 25 years, and is justly regarded as one of our most successful and accomplished physicians. The work of the health department for the year has been characterised by the same energy, vigilance and skilful application of sanitary science that has heretofore distinguished it under the present commissioner and the death-rate of Buffalo continues to be the lowest in the world among cities of its class.

The medical societies pursued their work on the usual lines and with reasonably progressive results. The Medical Society of the County of Erie held its customary two meetings during the year. The annual meeting, held January 12, 1897, was presided over by the retiring president, Dr. J. G. Thompson, of Angola. The following physicians were admitted to membership: Drs. Jane N. Frear, Frederick W. Hayes, Edward E. Koehler, Earl P. Lothrop, E. T. Rulison, A. E. Woehnert, Marion Marsh, Cora Billings Lattin and Henry W. Lattin. Dr. F. W. Hinckel read a paper on Earache, its significance and management, that has been extensively copied or abstracted by the medical journals throughout the country. Dr. P. W. Van Peyma read a paper on the Albuminuria of pregnancy and Dr. T. B. Carpenter one on Genito-urinary epithelium. At the semi-annual meeting, held June 8, 1897, Dr. Lucien Howe presided, having been elected president, vice Dr. H. R. Hopkins, declined. Dr. J. B. Coakley was elected vice-president, vice Dr. H. P. Trull, declined. Dr. J. Glen Ernest was elected to membership. Dr. William C. Krauss read a paper entitled Some facts regarding brain anatomy and brain tumors, and Drs. Frederick Preiss and R. R. Ross led in a discussion on the Application of the X-ray in medicine and surgery.

The Buffalo Academy of Medicine held its regular meetings, and some of the sections presented programs of unusual interest. The invitees from other cities to read papers, with the dates thereof, were as follows: Tuesday, January 12th, Dr. T. M. T. Finney, associate professor of surgery Johns Hopkins University Medical College, Baltimore, before the section on surgery— subject, Surgical treatment of perforating typhoid ulcer. February 2d, Dr. William B. Coley, surgeon to the Hospital for Ruptured and Crippled, New York, before the section on surgery—subject, Results of 350 operative cases of inguinal hernia in the male. Dr. William T. Councilman, professor of pathology in the Harvard Medical School, read a paper before the section on pathology, March 16th, entitled Acute diffuse nephritis, with special reference to the glomerula changes. Professor Charles Wright Dodge, of Rochester University, spoke before the section on pathology, April 20th, on the Recent progress in cellular biology, illustrated with the calcium light. Professor Burt G. Wilder, of Cornell University, delivered a lecture, May 18th, before the section on pathology on the Brain of a suicide, with fissural peculiarities. Dr. R. H. Harte, of Philadelphia, lectured to the section on surgery, June 29th—subject, Some observations on the radical cure of hernia. Dr. William H. Bergtold, professor of pathology in the University of Denver, Colorado, read a paper, September 28th, before the section on medicine, entitled Tuberculosis. Miss Mary Forster, Nawnham College, Eng., read before the section on pathology, September 21st—subject, Abnormal cell development in plants and animals: a study in comparative pathology. Dr. E. H. Bradford, surgeon to the Children's Hospital, Boston, spoke before the section on surgery, Thursday, November 4th—subject, Congenital dislocations of the hip, illus trated by lantern slides. Dr. John H. Musser, professor of clinical medicine University of Pennsylvania, lectured to the section of medicine, November 9th—subject, Renal calculus. Dr. William T. Corlett, professor of dermatology and syphilology Western Reserve University, Cleveland, spoke before the section on pathology, Tuesday, November 16th—subject, Recent researches in the pathology of trichophytosis, illustrated by stereopticon. The officers of the Academy, elected at the annual meeting in June, for 1897-98 are: President, Dr. Lucien Howe; secretary, Dr. Thomas F. Dwyer; treasurer, Dr. E. A. Smith; trustees, Dr. M. Hartwig, three years; Dr. Delancey Rochester, two years; and Dr. H. R. Hopkins, one year. The vice-presidents are the chairmen of the several sections. Section on medicine, chairman, Dr. Delancey Rochester; secretary, Dr. William G. Ring. Section on surgery, chairman, Dr. Chauncey P. Smith; secretary, Dr. A. E. Diehl. Section on obstetrics and gynecology, chairman, Dr. Charles Congdon; secretary, Dr. C. J. Reynolds. Section on pathology, chairman, Dr. F. J. Thornbury; secretary, Dr. J. C. Clemesha. The council consists of the foregoing officers and Dr. Herman Mynter, the president of the previous year.

The private medical societies have enjoyed a prosperous year, and many of them have done excellent work. The list is as follows: The Medical Club, the Medical Union, the Roswell Park Medical Club and the Buffalo Microscopical Society.

The American Institute of Homeopathy held its annual meeting at Buffalo, June 23–30, 1897. Dr. A. R. Wright, who acted as chairman of the local committee of arrangements, was elected president for the current year. The next meeting will be held at Omaha, Neb.

The Medical Association of Central New York held its annual meeting in this city, October 18, 1897, at the Ellicott Club, under the presidency of Dr. Edward B. Angell, of Rochester. A number of important papers were read and the medical expert question was discussed in a series of papers from both medical and legal standpoints. Mr. Tracy C. Becker, of Buffalo, presented an elaborate argument from the viewpoint of the bar. In this issue the Journal publishes a paper by Dr. A. W. Henckell, of Rochester, which opened the discussion. All the papers read at the meeting, after publication in the Journal, will appear in separate bookform.

The fifty-first annual commencement of Buffalo University Medical College was held April 27, 1897, when seventy diplomas were granted in medicine, forty-six in pharmacy and sixty-eight in dentistry, the largest number issued in any year. At the alumni association meeting Dr. P. W. Van Peyma, of Buffalo, presided, and, *inter alia*, Dr. Alfred Stengel, of Philadelphia, read an elaborate paper on the Anatomy and pathology of the red blood corpuscle. At the exercises held in the evening at Music Hall, Prof. Joseph P. Remington, of Philadelphia, addressed the graduating classes. The annual banquet was afterward held at the Ellicott Club.

The twelfth annual commencement of Niagara University Medical College was held May 12, 1897, at which ten diplomas were conferred. Dr. F. W. Maloney, of Rochester, presided at the meeting of the alumni association and also read a paper on the Importance of insurance medicine. Drs. Bayliss, Daggett and Dowd, of Buffalo, also read papers. At the evening exercises, Dr. John Cronyn acted as master of ceremonies and Dr. John A. Miller, Rev. Frank S. Fitch, of Buffalo, and the Rev. Father McHale, of Niagara Falls, delivered addresses. The customary banquet was served at the Genesee Hotel.

The Buffalo Sanatory Club was organised in February for the purpose of acquiring and disseminating knowledge relating to the preservation of health among the people. It aims to induce editors, reporters, microscopists, architects, builders, plumbers, heaters, men, women and doctors to join in the study and work aforementioned. It holds six meetings a year, at each one of which is discussed some topic relating to public health and prevention of disease.

The books of the year are: Appendicitis and its Surgical Treatment, by Dr. Herman Mynter, and an Epitomé of the History of Medicine, by Dr. Roswell Park. Dr. Mynter's treatise has been favorably received by the profession and has been commended by the medical journals. It is a most satisfactory exposé of our present knowledge of appendicitis. Dr. Park's work is a grouping of lectures delivered at the University of Buffalo, and is a most instructive and readable volume.

The dead of the year in Buffalo and vicinity are as follows: Dr. John C. Lewis, Panama, Chautauqua county, February 6, 1897, aged 48 years; Dr. Alphonse Dagenais, of Buffalo, March 4, 1897, aged 50 years; Dr. James B. Samo, of Buffalo, March 13th, aged 85 years; Dr. Frederic W. Bartlett, of Buffalo, March 17th, aged 71 years; Dr. Gardner C. Clarke, of Niagara Falls, March 22d, aged 56 years; Dr. Samuel Potter, of Lancaster, June 12th, aged 83 years ; Dr. F. H. James, of Lancaster, June 28th, aged 72 years ; Dr. Edward Storck, of Buffalo, July 26th, aged 66 years ; Dr. Myron H. Mills, of Mount Morris, August 14th, aged about 70 years ; Dr. Walter J. Ransom, of Lockport, August 20th, aged about 40 years ; Dr. George W. Lewis, of Buffalo, August 30th, aged 70 years ; Dr. Ravel B. Parks, of Jamestown, November 17th, aged 48 years.

